# Pubic Osteomyelitis After Laparoscopic Simple Prostatectomy: Pubic Bone Resection With Partial Cystectomy

**DOI:** 10.7759/cureus.53390

**Published:** 2024-02-01

**Authors:** João Aragão Vital, Miguel Marques Monteiro, José Silva Soares, Frederico Teves, Avelino Fraga

**Affiliations:** 1 Urology, Hospital Central do Funchal, Funchal, PRT; 2 Urology, Centro Hospitalar Universitário de Santo António, Porto, PRT

**Keywords:** pseudomonas aeruginosa, urinary tract infection, prostatectomy, benign prostatic hyperplasia, osteomyelitis

## Abstract

Osteomyelitis of the pubic symphysis presents a diagnostic challenge, characterized by symptoms of pubic pain and discomfort radiating to the groin, thigh, or hip. Post-prostate surgery occurrences are rare, with a propensity for cancer-related procedures. Conservative antibiotic therapy may prove insufficient, necessitating surgical intervention. This article details a unique case involving *Pseudomonas aeruginosa* infection, the second most prevalent pathogen. Despite the rarity of the diagnosis, particularly after a benign surgical procedure, timely intervention was hindered, leading to a delayed management course. The case involves a 69-year-old male with a history of benign prostatic hyperplasia who underwent laparoscopic simple prostatectomy. Post surgery, he developed recurrent urinary infection-related symptoms, leading to hospitalization. Diagnostic tools such as CT scans, MRI, and F-18-FDG-PET/CT scan played crucial roles in identifying the inflammatory process. Subsequent surgical debridement, pubic bone resection, and partial cystectomy, followed by an eight-week antibiotic course, led to a favorable recovery. Discussion emphasizes the rarity of pubic symphysis osteomyelitis, particularly after benign surgery, underscoring the importance of imaging and timely intervention. The presented case adds to the limited literature on post-prostatectomy osteomyelitis, emphasizing the need for heightened clinical awareness and consideration of rare complications even in routine surgical scenarios.

## Introduction

Osteomyelitis of the pubic symphysis, an intricate-to-diagnose infectious condition, manifests with symptoms like pubic pain and discomfort extending to the groin, thigh, or hip [1,2]. Complications after prostate surgery are rare, predominantly associated with cancer-related procedures [3-7]. Patients may exhibit limited response to conservative antibiotic treatment, necessitating surgical debridement [8]. This article presents a distinctive case characterized by *Pseudomonas aeruginosa* infection, the second most prevalent pathogen [9]. The atypical diagnosis, particularly following a benign surgical procedure, resulted in delayed intervention.

## Case presentation

A 69-year-old man with a history of benign prostatic hyperplasia (BPH) was referred to urology for refractory urinary retention. He had been taking tamsulosin for several years and had a previous history of elevated prostate-specific antigen (PSA) with three negative prostate biopsies.

During the evaluation, the patient had a bladder catheter in place, demonstrated prostatic enlargement in the digital rectal exam, a total PSA level of 10 ng/ml, and a Prostate Imaging-Reporting and Data System (PI-RADS) 2 score in the multiparametric MRI, estimating a prostate weight of 133 grams, and exhibited normal urinalysis parameters with a negative urine culture.

The patient underwent laparoscopic simple prostatectomy by transcapsular (Millin) approach, without intraoperative or post-surgical complication, with bladder catheter removal three days after surgery. The histopathology confirmed the diagnosis of BPH.

Five weeks after the procedure, the patient experienced pelvic and groin pain, radiating down the thigh, which was aggravated by sitting. There were no associated fever or lower urinary tract symptoms (LUTS). The patient presented to the emergency department and was diagnosed with a urinary tract infection (UTI) based on a positive urine culture for *Pseudomonas aeruginosa*. A CT scan revealed a 3.8 cm fluid collection in the Retzius space. The patient underwent a two-week course of meropenem antibiotic, which resulted in the resolution of symptoms. Following this initial episode, the patient experienced multiple relapses with recurring positive urine cultures for *Pseudomonas aeruginosa*, necessitating further cycles of antibiotics. Subsequently, a cystoscopy was performed, and no fistula was observed. Only some residual suture lines from the previous surgery were identified and subsequently removed during endoscopy.

**Figure 1:**
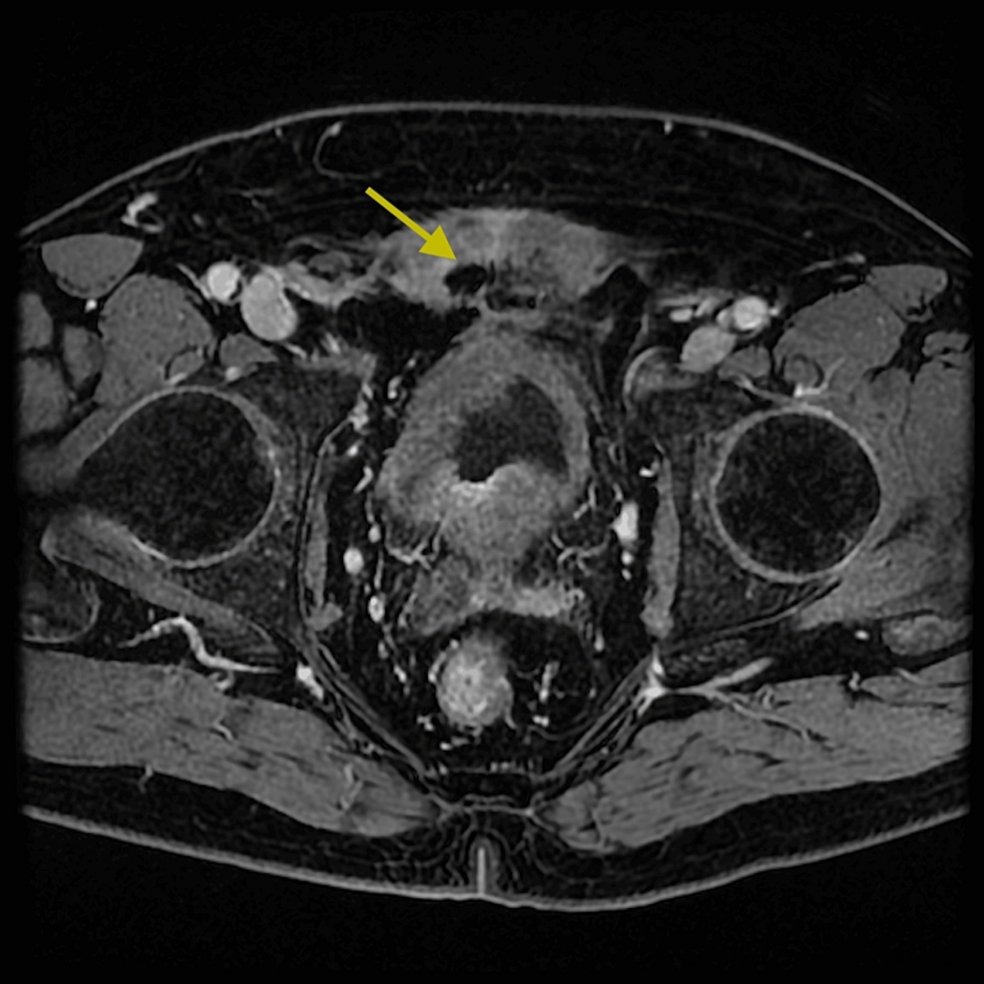
Axial pelvic MRI scan Multiple small abscesses within the Retzius space (arrow).

Six months following the surgical intervention, the patient's symptoms worsened, manifesting LUTS and severe pelvic pain that intensified during walking or movement. A positive urine culture indicated the presence of the same bacteria, leading to hospitalization and the initiation of targeted intravenous antibiotic treatment. A pelvic MRI scan revealed multiple small abscesses within the Retzius space, demonstrating apparent communication with the central defect resulting from the adenomectomy (Figure 1).

**Figure 2:**
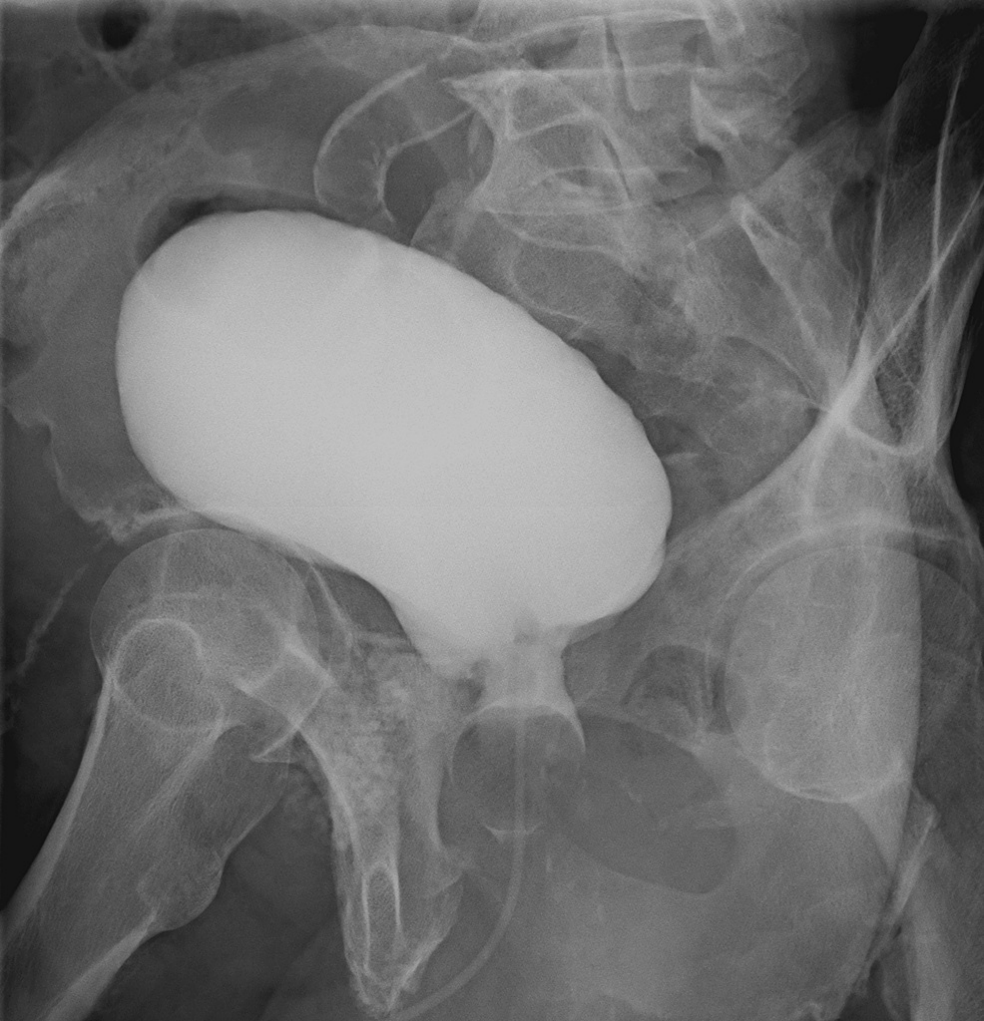
Postoperative cystogram (sagittal view) Observation of the shape of the bladder after surgery with instillation of 300 ml of fluid without leakage.

Additionally, signs of pubic osteomyelitis with extension to the rectus and obturator muscles bilaterally were observed. An F-18-FDG-PET/CT scan highlighted anomalous hypermetabolism in the pubic symphysis and surrounding tissues, including the external iliac and inguinal lymph nodes, suggestive of an ongoing inflammatory process. Subsequently, after more than two weeks of antibiotics, the patient underwent an exploratory laparotomy, during which surgical debridement, pubic bone resection, and partial cystectomy were performed. Histopathological examination confirmed the diagnosis, and the intra-surgical culture returned positive for *Pseudomonas aeruginosa*. The bladder catheter was removed three weeks post surgery, following a cystogram that showed no signs of leakage (Figure 2). He completed an eight-week course of antibiotics post operation. As of the present date, the patient has experienced a favorable recovery with no residual symptoms.

## Discussion

Osteomyelitis of the pubic symphysis is an uncommon infectious condition that is often challenging to diagnose [1]. The most observed symptoms are pain in the pubic area or discomfort that radiates to the groin, thigh, or hip. Involvement of the hip adductor muscles often results in pain while walking or during hip movement [2]. Genuine instances of osteomyelitis following prostate surgery are infrequently documented, with the majority occurring in the context of cancer-related surgical procedures [3-7]. In series reported, patients of this nature do not exhibit a positive response to conservative antibiotic therapy and may necessitate surgical debridement [8]. The syndrome of pubic osteomyelitis after prostate surgery remains underrecognized, and numerous individuals who experience recurring urinary infections, pelvic discomfort, and malaise may, in fact, have concealed osteomyelitis [8]. MRI excels in osteomyelitis detection, surpassing radiography and CT scans in both sensitivity and specificity [10]. Nuclear medicine studies have proven valuable in visualizing inflammatory processes and exhibit a high level of sensitivity in detecting osteomyelitis [11].

In prostate cancer treatment, pubic symphysis osteomyelitis with a urinary tract fistula is a rare but known complication [12]. We were unable to find a case report after a simple prostatectomy. In this case, we report a pubic symphysis osteomyelitis with a urinary tract fistula with *Pseudomonas aeruginosa* infection, the second most common pathogen in this pathology [9]. We cannot be certain whether the patient was already colonized at the time of the prostatectomy, despite a previous negative urine culture. In an unusual diagnosis, especially even rarer after a benign surgery, there was a delay in the proper management of this condition. The MRI played a crucial role in identifying it, and we utilized the F-18-FDG-PET/CT scan as an additional examination to enhance confidence. Surgical debridement, followed by extended antibiotic treatment and physiotherapy, was necessary and essential in resolving this severe complication.

## Conclusions

This case report highlights a rare and challenging case of pubic symphysis osteomyelitis with a urinary tract fistula following a benign laparoscopic simple prostatectomy. Careful intraoperative dissection, avoiding injury to the pubic bone, and ensuring a negative urine culture are crucial to avoid this complication. Diagnosis using advanced imaging techniques and surgical intervention, along with extended antibiotic therapy, were essential for a successful patient outcome. This case emphasizes the importance of considering rare complications even after routine surgical procedures.
